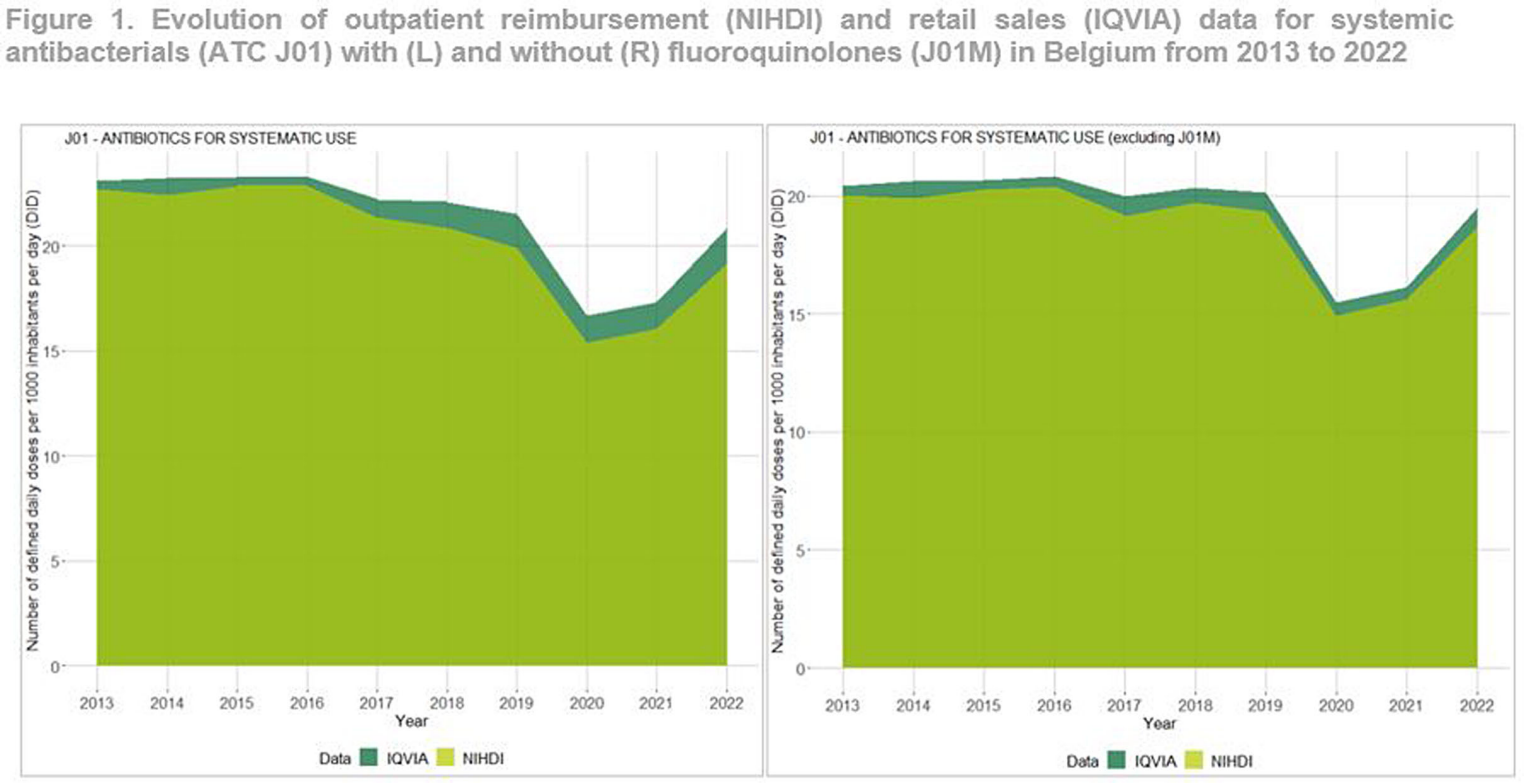# Outpatient Antibiotic Consumption Trends in Belgium: A Comparative Analysis of Reimbursement and Sales Data, 2013-2022

**DOI:** 10.1017/ash.2024.331

**Published:** 2024-09-16

**Authors:** Elena Damian, Laura Bonacini, Moira Kelly, Boudewijn Catry, Lucy Catteau

**Affiliations:** Sciensano; Department of Epidemiology and public health, Sciensano, Brussels, Belgium

## Abstract

**Background:** Antimicrobial resistance (AMR) is a global public health concern, necessitating close and timely monitoring of antibiotic consumption (AMC). In Belgium, AMC surveillance traditionally relies on reimbursement data, excluding over-the-counter non-reimbursed or imported products and involving a time lag. This study investigates disparities in AMC between reimbursement data and retail data, providing insights into AMC variations. Additionally this study seeks to critically evaluate the validity and representativeness of the reimbursed data in accurately reflecting the true extent of AMC in the country. **Method:** Utilizing reimbursement data from the National Institute for Health and Disability Insurance (NIHDI) and retail data (IQVIA Sales data; www.iqvia.com) for systemic antibacterials (ATC Group J01), outpatient consumption was estimated for the period 2013-2022. Volume of antimicrobials was measured in Defined Daily Doses (DDDs - WHO ATC/DDD Index 2023), while population data were extracted from Eurostat. Relative differences (RDs) in DDDs per 1000 inhabitants per day (DID) were computed, and validated through correlation analysis (Pearson’s r) and Bland–Altman plots. **Result:** J01 antibacterial sales declined from 23.10 DID (2013) to 20.85 (2022). Non-linear decreases, notably during the Covid-19 pandemic (21.54 DID in 2019 to 16.69 in 2020), followed by a rebound to pre-pandemic quantities in 2022 were observed (Figure 1). Reimbursement NIHDI data slightly underestimated IQVIA sales, with RDs ranging from 2% (2013) to 9% (2022). Notable differences, especially in recent years were attributed to quinolone reimbursement criteria changes implemented by law in Belgium in 2018, reducing the reimbursed proportion from 99% (2017) to 35% (2022). ATC-3 level analysis revealed disparities in low-DID groups (J01B, J01E and J01G). Notably, a small proportion of amphenicols (J01B) were reimbursed ( < 1 0%), with a congestion relieving combination product of tiamphenicol (+ N-acetylcysteine; Fluimucil®) frequently bought and remaining unreimbursed. Overall and across ATC3 groups, the correlation between NIDHI and IQVIA estimates was almost perfect across years and the Bland–Altman plots showed high agreement. **Conclusion:** Reimbursement data are reliable for outpatient AMC monitoring with slightly lower estimates than retail data across most categories. The 2018 quinolone reimbursement criteria change highlights the necessity of incorporating retail data for accurate assessments in this specific category. The synergistic use of reimbursement and retail datasets is crucial for a comprehensive understanding of consumption patterns, supporting effective AMR mitigation strategies in Belgium.

**Figure:**